# Identification and Characterization of a Novel Cathelicidin from *Hydrophis cyanocinctus* with Antimicrobial and Anti-Inflammatory Activity

**DOI:** 10.3390/molecules28052082

**Published:** 2023-02-23

**Authors:** Shuocun Wang, Liming Fan, Hanyu Pan, Yingying Li, Xin Zhao, Yan Qiu, Yiming Lu

**Affiliations:** 1 School of Medicine, Shanghai University, Shanghai 200444, China; 2 Department of Pharmacy, Shanghai Pudong New Area People’s Hospital, Shanghai 201299, China; 3 Department of Critical Care Medicine, Shanghai Tenth People’s Hospital, School of Medicine, Tongji University, Shanghai 200072, China

**Keywords:** bacterial resistance, Cathelicidin, antimicrobial peptide, sea snake, infection, bioinformatic prediction

## Abstract

The abuse of antibiotics and lack of new antibacterial drugs has led to the emergence of superbugs that raise fears of untreatable infections. The Cathelicidin family of antimicrobial peptide (AMP) with varying antibacterial activities and safety is considered to be a promising alternative to conventional antibiotics. In this study, we investigated a novel Cathelicidin peptide named Hydrostatin-AMP2 from the sea snake *Hydrophis cyanocinctus*. The peptide was identified based on gene functional annotation of the *H. cyanocinctus* genome and bioinformatic prediction. Hydrostatin-AMP2 showed excellent antimicrobial activity against both Gram-positive and Gram-negative bacteria, including standard and clinical Ampicillin-resistant strains. The results of the bacterial killing kinetic assay demonstrated that Hydrostatin-AMP2 had faster antimicrobial action than Ampicillin. Meanwhile, Hydrostatin-AMP2 exhibited significant anti-biofilm activity including inhibition and eradication. It also showed a low propensity to induce resistance as well as low cytotoxicity and hemolytic activity. Notably, Hydrostatin-AMP2 apparently decreased the production of pro-inflammatory cytokines in the LPS-induced RAW264.7 cell model. To sum up, these findings indicate that Hydrostatin-AMP2 is a potential peptide candidate for the development of new-generation antimicrobial drugs fighting against antibiotic-resistant bacterial infections.

## 1. Introduction

Antibiotics represented by penicillin are great inventions that fight bacterial infections and have saved thousands of lives [1]. However, the abuse of antibiotics in recent years has given rise to the emergence of antimicrobial resistance (AMR), which poses one of the greatest threats to human health. In 2019 [2], 1.27 million people died directly from AMR and 4.95 million deaths were related to drug-resistant bacterial infections. To address this issue, global researchers are making efforts toward the development of new antibacterial drugs, among which AMP are considered to be promising substitutes for traditional antibiotics and have aroused wide public concern [3].

AMP are critical defensive molecules in the innate immune systems of most organisms. Among them, linear helical peptides without cysteine constitute the largest group [4]. Boman [5] first discovered a 37-mer AMP, α-helical, amphiphilic, and cationic, named Cecropin in silkworm pupae, which acts directly on the bacterial cell membrane to produce its bactericidal effect. Magainin [6], a 22-mer cationic helical peptide from the skin of Xenopus, kills bacteria by permeabilizing the cell membrane without exhibiting significant toxicity against mammalian cells. It had reached the U.S. Food and Drug Administration (FDA) standard to reduce clinical infectious symptoms of diabetic foot ulcers. Most importantly, cationic AMP works in a way distinctly different from conventional antibiotics, which hardly induce drug resistance. By electrostatic interaction, positively charged AMP are absorbed by bacterial membranes [7]. Hydrophilic residues act on the phospholipid head molecules and insert into the membrane. The interaction causes changes of the permeability of the membrane so that intracellular ions and metabolites leak out and finally the cell membrane cracks. Cathelicidin is a key family of helical AMP which is found only in vertebrates. The first Cathelicidin isolated from humans, LL-37 [8], has entered clinical studies (NCT04098562, NCT02225366). The precursor proteins of Cathelicidins have conserved N-terminal signaling peptides and the cathelin region, but the mature peptides at the C-terminal are highly variable [9]. Cathelicidin peptides were reported to have not only antimicrobial functions against bacteria, fungi, viruses and even parasites, but also immunomodulatory functions such as chemotaxis and activation of immune cells [10], inhibition of NADPH oxidase, promotion of angiogenesis [11] and wound healing [12]. Therefore, Cathelicidins are important candidates for anti-infective drug development [13,14].

Marine organisms are natural sources of a variety of bioactive molecules [15]. *H. cyanocinctus* is an ancient sea snake that has long been used in traditional Chinese medicine and modern biopharmaceuticals for medicinal components [16]. Our group [17] has previously sequenced and assembled the high-quality genome of *H. cyanocinctus*. In this study, we identified a novel Cathelicidin Hydrostatin-AMP2 in the genome of *H. cyanocinctus.* The potential antimicrobial activity of Hydrostatin-AMP2 was characterized by a bioinformatic algorithm and multiple sequence alignment. Further studies proved that Hydrostatin-AMP2 had not only a wide range of bacteriostasis, fast bactericidal speed, and the ability to inhibit and eradicate biofilms, but also significant anti-inflammatory activity.

## 2. Results

### 2.1. Identification of Hydrostatin-AMP2

**Figure 1:**
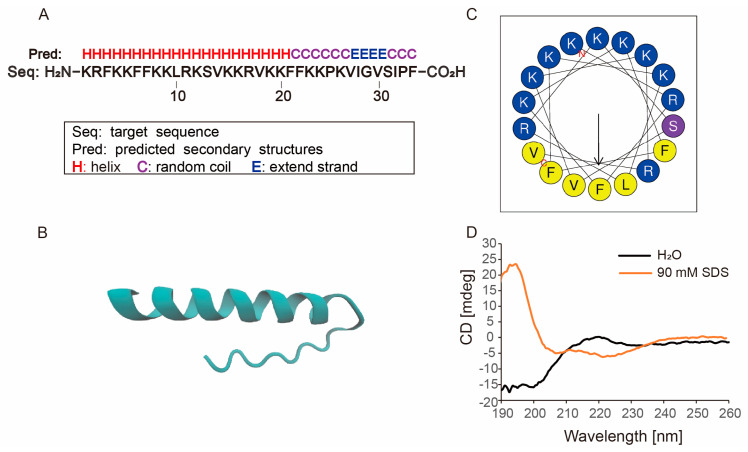
Structure characterization of Hydrostatin-AMP2. (**A**) Primary and secondary structure of Hydrostatin-AMP2; (**B**)Three-dimensional structure of Hydrostatin-AMP2 predicted by QUARK; (**C**) Helix-wheel diagram of the represented α-helix region of Hydrostatin-AMP2; (**D**) Circular dichroism spectra of Hydrostatin-AMP2 in H_2_O and 90 mM sodium dodecyl sulfate (SDS).

To identify the antimicrobial region from the *H. cyanocinctus* genome, many bioinformatics tools were used. The potential Cathelicidin genes were identified in the *H. cyanocinctus* genome using the blast with protein database. Predictions of conservative domains in the Cathelicidin family were made by the Conserved Domains module in the National Center of Biotechnology Information (NCBI). AMPA server [18] was used to predict antimicrobial regions. CAMPR3 [19] and DBAASP (databases of antimicrobial activity and structure of peptides) [20] were used to predict antimicrobial activities. Finally, a new Cathelicidin (mature peptide) was discovered and named Hydrostatin-AMP2. It was predicted to be an amphiphilic cationic polypeptide with antibacterial activity against Gram-negative bacteria and little hemolytic activity. The physical and chemical properties of Hydrostatin-AMP2 were analyzed by ProtParam [21]. Hydrostatin-AMP2 contains 34 amino acid residues (Figure 1A) with a sequence of KRFKKFFKKLRKSVKKRVKKFFKKPKVIGVSIPF. It is composed of 9 types of amino acids, in which lysine and phenylalanine residues account for the highest proportion of 38.2% and 17.6%, respectively. There are no negatively charged amino acid residues but 16 positively charged residues (arginines and lysines) in the sequence. The molecular weight is computed to be 4197.35 Da (C_206_H_344_N_56_O_37_) and the theoretical isoelectric point is 12.34. The instability index (II) is 17.20 and the grand average of hydropathicity (GRAVY) is −0.676, indicating that Hydrostatin-AMP2 is a stable hydrophilic cationic polypeptide.

### 2.2. Molecular Structure of Hydrostatin-AMP2

In order to investigate the possible structure-function relationship, the secondary and tertiary structure of Hydrostatin-AMP2 was predicted by QUARK [22] (Figure 1B). The peptide displayed an α-helix structure at the N-terminal region and random coil at the C-terminus. Then the HeliQuest server [23] was used to plot the N-terminal helical wheel diagram of Hydrostatin-AMP2 (Figure 1C). There are clear distribution boundaries between hydrophobic amino acids (shown in yellow) and hydrophilic amino acids (shown in blue and purple). This amphiphilic distribution is related to the mode of action of AMP. It is generally believed that cationic amino acids (shown in blue) are attracted to the surface of the anionic membrane of bacteria through electrostatic adsorption, and hydrophobic amino acids gather on the side to form pores, thus disturbing the bacterial membrane and killing bacteria.

To verify the accuracy of the predicted structure, the native secondary structure of Hydrostatin-AMP2 in H_2_O and 90 mM SDS solution was determined by circular dichroism (CD). Hydrostatin-AMP2 exhibited a random coil structure in H_2_O with a negative absorption peak at 200 nm (Figure 1D). In the 90 mM SDS pseudo-membrane environment, the CD spectrum of Hydrostatin-AMP2 forms a typical α-helical positive peak near 192 nm. These results indicate that Hydrostatin-AMP2 functions mainly in an α-helix conformation in hydrophobic or pseudo-membrane environments.

### 2.3. Sequence Comparison and Phylogenetic Analysis

**Figure 2:**
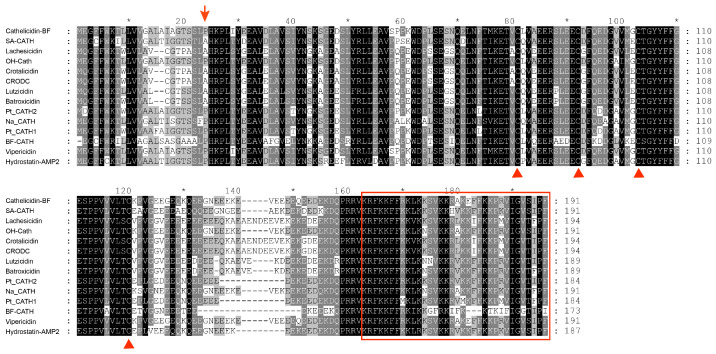
Multi-sequence alignment of Hydrostatin-AMP2 with other snake Cathelicidins. Sequences retrieved from NCBI were multi-aligned with Clustal Omega. Dashes were inserted to optimize the alignment. Highly conserved residues were shaded and identical residues were indicated in black. Arrows indicated the boundary between the signal region and the cathelin domain. The four cysteine positions at the end of the cathelin domain were indicated by triangles. The C-terminal region (mature peptide) where the antimicrobial activity resides was boxed.

We performed multiple sequence alignments between the Hydrostatin-AMP2 precursor and other representative snake Cathelicidins. These Cathelicidin precursors consistently consist of three parts including the N-terminal signal peptide sequence, the highly conserved cathelin domain, and the highly heterogeneous C-terminal mature peptide sequence (antimicrobial region). The comparison results showed that the Hydrostatin-AMP2 precursor was overall homologous to other snake Cathelicidins (Figure 2). The four cysteine positions at the end of the cathelin domain were identical. Hydrostatin-AMP2 precursor had a homology of 80.6% with Na-CATH, and the homology between Hydrostatin-AMP2 and the mature Cathelicidin peptides of other snakes was 91.18% with BF-CATH, 88.57% with OH-CATH, and 85.71% with Na-CATH. Among them, Hydrostatin-AMP2 differs from other mature peptides mainly in arginine 11, valine 18, and lysine 26. Arginines and lysines are alkaline amino acids that can increase the positive charges of peptides. The Positive charge is closely related to electrostatic interaction between peptide and microbe. Meanwhile, as hydrophobic amino acid, valine can promote the interaction between peptides and microbial membranes to disturb membranous structure. As a result, this difference affected by arginine 11, valine 18 and lysine 26 may give Hydrostatin-AMP2 greater biological activity.

**Figure 3:**
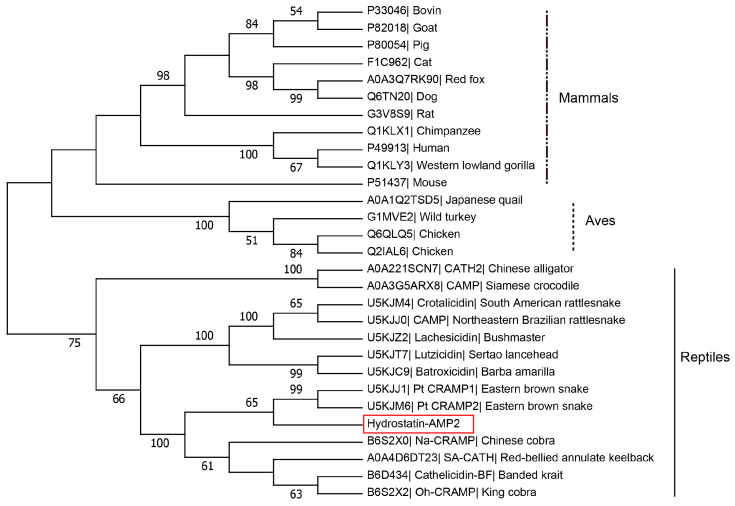
Molecular phylogenetic analysis of Hydrostatin-AMP2. The tree was obtained by neighbor-joining analysis based on the proportional difference of the signal peptide and cathelin domain sequence of Cathelicidins. Only bootstrap values >50% (expressed as percentages of 1000 resamplings) were shown. Hydrostatin-AMP2 was boxed.

We also performed a phylogenetic analysis of Hydrostatin-AMP2 and other representative Cathelicidins of mammals, aves, and reptiles (Figure 3). Hydrostatin-AMP2 clustered with the Cathelicidins of terrestrial snakes, implying that Hydrostatin-AMP2 and the Cathelicidins of terrestrial snakes are from the same ancestor. In addition, Cathelicidins from elapids and vipers clearly formed two distinct subclusters, with Hydrostatin-AMP2 clustering with elapid Cathelicidins, suggesting that Hydrostatin-AMP2 is more closely related to elapid Cathelicidins.

### 2.4. Antimicrobial Activities of Hydrostatin-AMP2

In order to evaluate the antibacterial activities of Hydrostatin-AMP2, we measured its minimal inhibitory concentration (MIC) and minimum bactericidal concentration (MBC) against different bacteria with Ampicillin as the positive control. Hydrostatin-AMP2 showed inhibitory activity against six strains of Gram-negative strains and two strains of Gram-positive strains tested with the MIC ranging from 16 to 32 μg/mL (Table 1). Among the eight tested strains, *Escherichia coli* was the most sensitive with a MIC value of 16 μg/mL. It is noteworthy that five strains of *Klebsiella pneumoniae* showed Ampicillin resistance. This reflects the potential of Hydrostatin-AMP2 as a drug in clinical treatment against drug-resistant microorganisms.

### 2.5. Time-Kill Kinetics of Hydrostatin-AMP2

**Figure 4:**
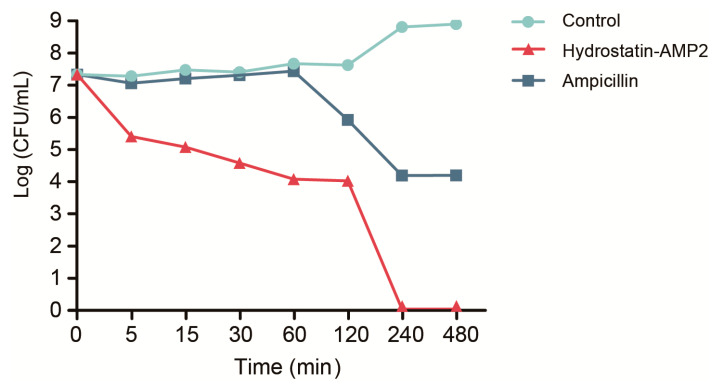
Killing kinetics of Hydrostatin-AMP2 against *E. coli*. After incubation with Hydrostatin-AMP2 or Ampicillin for a different time, CFU was determined by serial dilution agar plating method. Data represent mean ± SD values of three independent experiments performed in triplicate.

In order to test the bactericidal rate of Hydrostatin-AMP2, colony forming unit (CFU) measurement was used to determine the bactericidal kinetics. Hydrostatin-AMP2 showed rapid bactericidal activity (Figure 4) against *E. coli* at 32 μg/mL (MBC). The peptide began to show killing effect for *E. coli* within 5 min and the bactericidal rate was up to 29%. After 2 h, Hydrostatin-AMP2 have killed bacteria about 57%. After 4 h, the bacteria were killed completely. In contrast, Ampicillin (MBC) started to display a bacteriostatic effect at least after 1 h. After being co-incubated for 4 h, the killing rate was about 47% which was lower than Hydrostatin-AMP2 within the first 2 h. In addition, the effect of Ampicillin didn’t have any improvement from 4 h to 8 h. Ampicillin failed to completely kill *E. coli* within 8 h.

### 2.6. Inhibition and Eradication of Biofilms by Hydrostatin-AMP2

**Figure 5:**
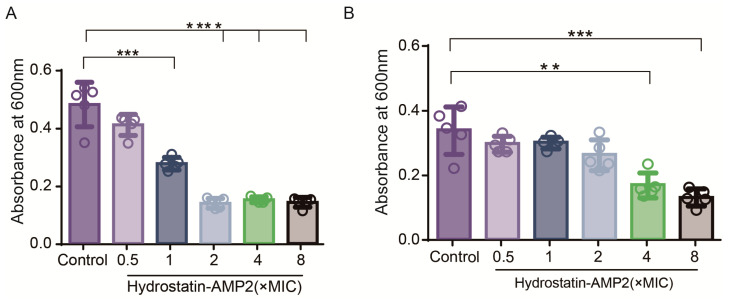
Effects of Hydrostatin-AMP2 on *S. aureus* biofilm. (**A**) Biofilm inhibition activity of Hydrostatin-AMP2. (**B**) Biofilm eradication activity of Hydrostatin-AMP2. Data represent mean ± SD values of three independent experiments performed in quintuplicate. **, *p* < 0.01; ***, *p* < 0.001; ****, *p* < 0.0001 (significantly different compared with the control).

Biofilms are communities of microorganisms that attach to the surface of objects and can increase bacterial resistance. *Staphylococcus aureus* is a common standard strain in the study of biofilm which has a strong ability to form biofilm. Therefore, we studied the inhibition effect of Hydrostatin-AMP2 on biofilm formation and its eliminating effect on mature biofilms of *S. aureus* by crystal violet staining and quantitative analysis. The inhibition of Hydrostatin-AMP2 on the biofilm formation of *S. aureus* reached 71.02% at the concentration of 2 × MIC (Figure 5A). When the peptide concentration changed from 4 × MIC to 8 × MIC, the scavenging effect of Hydrostatin-AMP2 on mature biofilms increased from 50.02% to 60.99% (Figure 5B). These results indicated that Hydrostatin-AMP2 demonstrated strong inhibitory and eradicative activity against the biofilms of *S. aureus* in a dose-dependent manner.

### 2.7. Induction of Drug-Resistance

**Figure 6:**
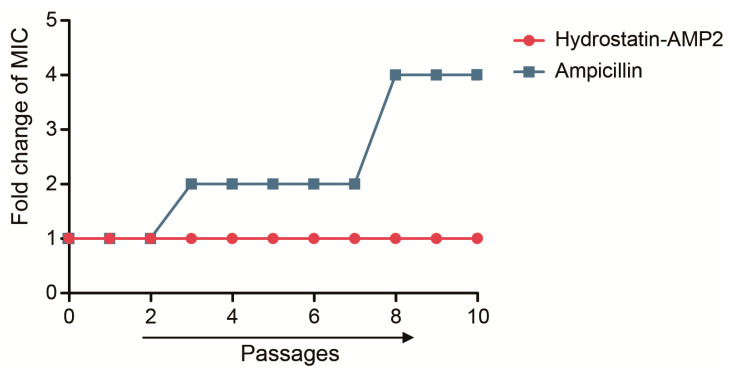
Hydrostatin-AMP2 did not induce significant drug resistance in *E. coli*. Resistance was determined by comparing the change of MIC value of *E. coli* to Hydrostatin-AMP2 and Ampicillin after 10 passages. Data represent mean ± SD values of three independent experiments performed in triplicate.

We mixed Hydrostatin-AMP2 or Ampicillin at the concentration of 0.5 × MIC with the growing cells of *E. coli* and investigated the ability of induction of drug resistance. After 10 consecutive days of induction, the MIC value of Ampicillin was quadrupled while the sensitivity of *E. coli* to Hydrostatin-AMP2 remained unchanged (Figure 6), indicating that the peptide would not induce obvious bacterial resistance under established conditions.

### 2.8. Salt Tolerance, Thermal Tolerance, pH Stability and Serum Stability of Hydrostatin-AMP2

We examined the activity of Hydrostatin-AMP2 under different physical and chemical conditions. Previous studies suggested that salt could significantly antagonize AMP and affect their activity [24]. Therefore, this study investigated the impact of different concentrations of NaCl or CaCl_2_ solution on the activity of AMP according to the shift of MIC against *E. coli*. The MIC value of Hydrostatin-AMP2 merely doubled from 16 to 32 μg/mL (Table 2) with the NaCl concentration increasing from 0 to 150 mM. When the NaCl concentration reached 200 mM, this effect is consistent with CaCl_2_ under physiological concentration and extreme pH.

We also tested the change in the MIC value of Hydrostatin-AMP2 after incubation at different temperatures. Hydrostatin-AMP2 activity remained stable over a wide range of 4 to 80 °C (Table 2). In addition, the antibacterial capability of Hydrostatin-AMP2 was still maintained with an MIC value of 32 μg/mL after incubation with serum for 4 h (Table 2).

### 2.9. Hemolytic Activity and Cytotoxicity of Hydrostatin-AMP2

Erythrocytes, L929, RAW264.7 and HaCaT cells were used to determine the possible hemolysis activity and cytotoxicity of Hydrostatin-AMP2 [25]. Hydrostatin-AMP2 yielded a hemolysis rate of about 4.6% on blood cells at 250 μg/mL (Table 3). Toxicity studies showed that Hydrostatin-AMP2 induced cell death percentages of lower than 10% on the tested cell lines at the concentration of 32 μg/mL. Therefore, the peptide displayed relatively low cytotoxicity within its dose range of antimicrobial activity.

### 2.10. Hydrostatin-AMP2 Inhibited the Pro-Inflammatory Factor Expression Induced by LPS

**Figure 7:**
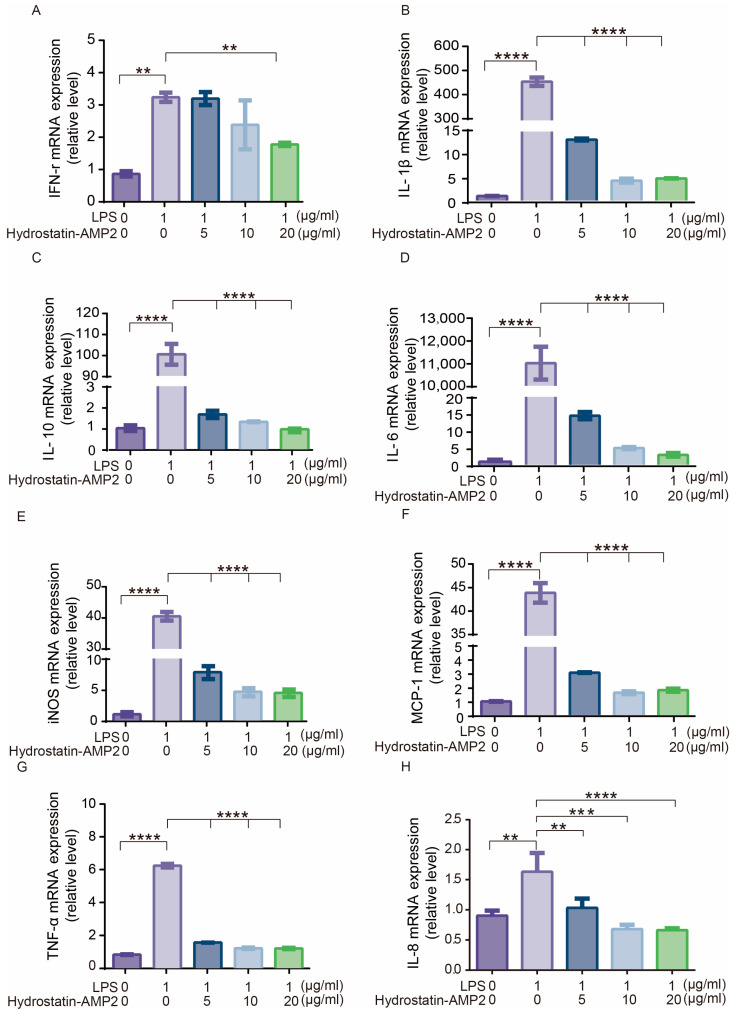
Effects of Hydrostatin-AMP2 on LPS-induced expression of pro-inflammatory factors. (**A**–**H**) Relative mRNA expression level of IFN-γ, IL-1β, IL-10, IL-6, iNOS, MCP-1, TNF-α and IL-8. Data represent mean ± SD values of three independent experiments performed in triplicate. **, *p* < 0.01; ***, *p* < 0.001; ****, *p* < 0.0001 (significantly different compared with the model group).

Lipopolysaccharide (LPS), also known as endotoxin, is a component of the outer wall of Gram-negative bacteria. When the cell wall of Gram-negative bacteria is disturbed, LPS released. LPS can cause a series of inflammatory reactions in organisms, and can even lead to sepsis and endotoxic shock. In this study, we used the LPS-induced RAW264.7 cell model to analyze the effect of Hydrostatin-AMP2 on the expression of inflammatory factors. The mRNA expression of interleukins (IL-6, IL-1β, IL-10, IL-8), tumor necrosis factor-alpha (TNF-α), interferon-gamma (IFN-γ), monocyte chemoattractant protein-1 (MCP-1) and inducible nitric oxide synthase (iNOS) in the model group was obviously increased (Figure 7). Treatment with Hydrostatin-AMP2 significantly decreased the transcription of pro-inflammatory mediators and anti-inflammatory factors. Particularly, 20 μg/mL of Hydrostatin-AMP2 could reduce iNOS expression by 87.5%. Moreover, the inhibitory effect of Hydrostatin-AMP2 was dose-dependent within the range of 5–20 μg/mL.

## 3. Discussion

With the unceasing emergence of new drug-resistant microorganisms, AMP are seen as a potential alternative to restrain the rapid spread of antibiotic resistance. In recent years, bioinformatics has become an important vehicle to discover new AMP [26,27]. In this study, a novel cationic AMP named Hydrostatin-AMP2 was identified by mining the genome of a sea snake. Hydrostatin-AMP2 was an amphiphilic peptide that had disordered conformations in an aqueous solution but formed α-helices only in the membrane-mimetic environment. This was consistent with previous studies showing that the amphiphilic surfaces could perturb the structure of bacterial biofilms after the formation of helical conformations and then rapidly kill the bacteria [28]. Multiple sequence alignments displayed that Hydrostatin-AMP2 had high homology with other Cathelicidin peptides derived from snakes. The cathelin region and the four cysteine sites at the cathelin terminal were consistent, which suggested that Cathelicidin peptides are extremely conserved during evolution and may have important functions in snakes, such as innate immunity against pathogen infection and immune protection. In particular, the sequence of Hydrostatin-AMP2 was most similar to the mature peptide sequences from Elapidae snakes including *Bungarus fasciatus*, *Naja atra*, and *Ophiophagus hannah*, which accorded with their close evolutionary relationship with sea snakes previously reported by Li [17]. They sequenced the genome of the sea snake *H. cyanocinctus* and the phylogenetic analysis confirmed that this sea snake from Hydrophiinae also belongs to Elapidae. Accordingly, since the relationship was relatively distant between *H. cyanocinctus* and vipers such as *Lachesis muta rhombeata*, there existed a larger sequence difference between Hydrostatin-AMP2 and Lachesicidin. Moreover, the difference between Hydrostatin-AMP2 with other peptides is mainly in lysine and valine. Hydrostatin-AMP2 may possess a unique local structure by the differential amino acid.

Hydrostatin-AMP2 exhibited antimicrobial activities against Gram-positive and Gram-negative bacteria, the latter of which included *E. coli* and *K. pneumoniae*. Notably, the clinically isolated *K. pneumoniae* strains were not sensitive to Ampicillin. When the concentration of Ampicillin was 128 μg/mL, the bacterial growth was still not inhibited, but Hydrostatin-AMP2 showed significant inhibitory activity against *K. pneumoniae* at 16–32 μg/mL. This indicated that Hydrostatin-AMP2 had the potential to fight against drug-resistant bacteria. In the bacterial killing kinetic assay, Hydrostatin-AMP2 completely eliminated the bacteria within 4 h, and there was still no bacterial growth within 8 h. However, the bacterial growth could be partly maintained after co-incubation with Ampicillin for 4 h. Previous studies reported [29] that antibacterial peptides adsorbed on the cell surface by electrostatic action, disturbed the membrane structure, changed membrane permeability, and result in cell lysis. This way kills faster than effects with cell metabolism. Therefore, we suggested that Hydrostatin-AMP2 kills bacteria targeting cell membranes. This advantage of the mechanism gives AMP research potential. Therefore, Hydrostatin-AMP2 had not only a broad antibacterial spectrum, but also rapid and effective bactericidal effects as well as the ability to overcome drug resistance.

Biofilms are formed by a large number of bacteria adhering to the surface of objects and secreting proteins, polysaccharides, and other substances. Biofilms can encrust bacteria and build a protective barrier to reduce the effectiveness of antibiotics and induce resistance. Hydrostatin-AMP2 had dose-dependent inhibitory and scavenging activities against bacterial biofilms. After co-incubation with *S. aureus*, the inhibition efficiency on biofilm formation reached 77.70%. Furthermore, for mature biofilms that are already formed, Hydrostatin-AMP2 could achieve the highest eradication rate of 77.85% at 8 × MIC, which was similar to the efficacy of a modified peptide ZY4 reported previously [30].

The degradation rates and activities of AMP can be affected by different physical and chemical factors in the environment. We determined the MIC changes of Hydrostatin-AMP2 against *E. coli* under different conditions. The MIC value was 16–64 μg/mL under different salinity, extreme pH, and temperatures tested. Additionally, the MIC value of the peptide remained less than 32 μg/mL after serum treatment for 4 h. These observations indicated that Hydrostatin-AMP2 had strong salt tolerance, thermal tolerance, pH stability, and serum stability, which is suitable for application in various environments.

Considering the potential toxicity of AMP, we further investigated the cytotoxicity and hemolysis of Hydrostatin-AMP2. It has been reported that the hemolytic activity of OH-CATH and FP-CATH at 200 μg/mL was 10.80% and 16.13%, respectively. However, the hemolysis of Hydrostatin-AMP2 was only 4.6% at 250 μg/mL, suggesting that Hydrostatin-AMP2 had very low hemolysis activity. Moreover, the cytotoxic effect of Hydrostatin-AMP2 on a human epidermal cell line and two murine cell lines was less than 10% within the active concentration range, indicating that Hydrostatin-AMP2 also had little cytotoxicity.

The results of real-time PCR showed that 5–20 μg/mL Hydrostatin-AMP2 significantly inhibited the expression of pro-inflammatory factors (TNF-α, IL-8, IL-6, IL-1β, IFN-γ, etc.) in RAW264.7 cells induced by LPS. This anti-inflammatory action was considered to be associated with LPS-activated downstream signal pathways. In the innate immune mechanism of host defense against pathogenic microorganisms, LPS from Gram-negative bacteria can bind to Toll-like receptor 4 (TLR4), a transmembrane protein expressed on a variety of immune cells including RAW264.7 macrophages. LPS-TLR4 interaction would activate the Nuclear Factor-Kappa B (NF-κB) signaling pathway to transmit inflammatory signals, in which phosphorylated NF-κB enters the nucleus and binds to related target DNA to promote the gene transcription of downstream inflammatory cytokines (TNF-α, IL-8, IL-6, IL-1β, IFN-γ, etc.) [31,32,33]. In addition, high levels of TNF-α, IFN-γ, and LPS could promote the generation of MCP-1 and iNOS, and further activate the NF-κB pathway, resulting in a positive feedback effect and inflammatory cascade reaction [34,35] which lead to the continuous amplification and persistence of inflammation. As an immunomodulating response, IFN-γ can also induce regulatory T (Treg) cells and T helper 2 (Th2) cells to produce anti-inflammatory factors such as IL-10, which could to some degree represent the inflammation level in vitro. Therefore, we proposed that Hydrostatin-AMP2 inhibit the binding of LPS to TLR4 receptor or block NF-κB/iNOS signal transduction to play anti-inflammatory activity. It is noteworthy that AMP may kill bacteria in various ways [36]. As Hydrostatin-AMP2 exerted rapid bactericidal effects and anti-inflammatory effects, it might affect both ways involving membrane permeability and intracellular metabolism. This mechanism of action needs to be clarified in subsequent studies.

As a marine-derived bioactive peptide from the sea snake *H. cyanocinctus*, Hydrostatin-AMP2 can not only inhibit the growth of bacteria and the formation of biofilms, but also eradicate the formed biofilms. It shows rapid bactericidal activity and anti-inflammatory capacity as well as high stability and low toxicity. In conclusion, our study demonstrated the potency of Hydrostatin-AMP2 as a promising candidate for the development of novel antimicrobial drugs for the treatment of antibiotic-resistant infections, though structural modification and optimization are still needed to further develop its medicinal potential.

## 4. Materials and Methods

### 4.1. Identification and Activity Prediction of Hydrostatin-AMP2

The genome of sea snake *Hydrophis cyanocinctus* in the National Center for Biotechnology Information (NCBI, genome ID: 75161) was blasted with NCBI NR (release 2022_02, https://ftp.ncbi.nlm.nih.gov/blast/db/FASTA/nr.gz, accessed on 5 April 2022), Swiss-Prot (release 2022_03, https://www.uniprot.org/, accessed on 10 April 2022) and CAMPR3 (release 2021_10, http://www.camp.bicnirrh.res.in/, accessed on 20 April 2022) to perform gene function annotation. According to the annotated information, the target gene sequence was translated into amino acids, and its terminal was determined by AMPA (https://tcoffee.crg.eu/apps/ampa/do, accessed on 3 May 2022), CAMPR3 (http://www.camp3.bicnirrh.res.in/, accessed on 6 May 2022), and DBAASP server (https://dbaasp.org/home, accessed on 8 May 2022). The physical and chemical properties of peptide were analyzed through ExPASy (https://web.expasy.org/protparam/, accessed on 15 May 2022). The helix-wheel structures were constructed by HeliQuest (https://heliquest.ipmc.cnrs.fr/, accessed on 17 May 2022). Three-dimensional structure was obtained by QUARK (https://zhanggroup.org/QUARK/, accessed on 21 May 2022).

### 4.2. Multi-Sequence Alignment and Phylogenetic Analysis

Cathelicidin sequences were obtained from the NCBI database. Multiple sequence alignment was performed and visualized using the Clustal Omega web server (https://www.ebi.ac.uk/Tools/msa/clustalo/, accessed on 6 June 2022) [37] and GeneDoc v2.7.0.0 [38], respectively. Phylogenetic analysis was taken by MEGA 11 [39].

### 4.3. Biological Materials and Reagents

Hydrostatin-AMP2 was synthesized by Scilight-Peptide Inc. (Beijing, China) with a purity greater than 95%. The peptides were kept under refrigeration at −20 °C until use. The purity and molecular mass of the peptides were confirmed by high-performance liquid chromatography (Varian Prostar 218 HPLC Systems, Waltham, MA, USA) and mass spectrometric (Voyager-DE STR MS, Milford, MA, USA), respectively.

RAW264.7 (murine macrophage cell line), L929 (mouse fibroblast cell line), and HaCaT (human immortal keratinocyte cell line) were purchased from the Shanghai Institute of Biochemistry and Cell Science, Chinese Academy of Sciences.

The microorganisms (*Staphylococcus aureus* [ATCC 25923], *Escherichia coli* [ATCC 25922], and *Propionibacterium acnes* [ATCC 6919]) were obtained from Fuxiang Biotechnology (Shanghai, China). The five strains of *K. pneumoniae* clinical isolates (44, 48, 49, 50, and 51) were obtained from the Department of Critical Care Medicine, Shanghai Tenth People’s Hospital.

Sodium dodecyl sulfate (SDS) was purchased from Solarbio Science & Technology Co., (Beijing, China). Mueller-Hinton broth (MH) was purchased from Qingdao Hi-Tech Industrial Park Hope Bio-Technology Co., Ltd. (Qingdao, China). Ampicillin was purchased from Shanghai Macklin Biochemical Co., Ltd. (Shanghai, China). Methanol and anhydrous ethanol were purchased from Sensi Chemical Co., Ltd. (Shanghai, China). Crystal violet solution was purchased from Sangon Biotech Co., Ltd. (Shanghai, China). NaCl and CaCl_2_ were purchased from Shanghai Aladdin Biochemical Technology Co., Ltd. (Shanghai, China). Human serum and cell counting Kit-8 reagent were purchased from Kingmorn Biotechnology Co., Ltd. (Shanghai, China). Human red blood cells were purchased from Rockland Immunochemicals, Inc (Pottstown, PA, USA). Triton X-100 solution was purchased from Beyotime Biotechnology Co., Ltd. (Shanghai, China). Dulbecco’s Modified Eagle Medium (DMEM) and Fetal Bovine Serum (FBS) were purchased from Hyclone (Shanghai, China). Penicillin-streptomycin solution was purchased from Procell Life Science & Technology Co., Ltd. (Wuhan, China). Lipopolysaccharides from *E. coli* O111:B4 (LPS) were purchased from Sigma-Aldrich Trading Co., Ltd. (Shanghai, China). RNAiso Plus, TB Green^®^ Premix Ex Taq™ II, and PrimeScript™ RT Master Mix were purchased from Beijing Takara Biomedical Technology Co., Ltd. (Beijing, China). All chemicals and reagents used in this study were of analytical grade.

### 4.4. Circular Dichroism Spectroscopy

The secondary structure of Hydrostatin-AMP2 was evaluated by circular dichroism spectroscopy (CD, DHS International CO., LTD, MOS-500). Samples were prepared by dissolving in H_2_O and 90 mM SDS solutions. Measuring spectra at 190–260 nm. The instrument parameters are as follows: path-length cell (0.1 cm); bandwidth (1 nm); response time (1 s); scanspeed (100 nm/min).

### 4.5. Antimicrobial Assays

The antimicrobial assay was performed by broth microdilution method, following the guidelines of the Clinical and Laboratory Standards Institute [40,41]. Strains (mentioned in Section 4.3) were inoculated into Mueller-Hinton broth (MH), oscillated (37 °C, 220 rpm, 12 h) to the logarithmic growth stage, and then diluted to 10^6^ CFU/mL. In 96-well plates, 100 µL of Hydrostatin-AMP2 (1–256 μg/mL) in MH broth was incubated with 100 µL of the bacterial suspension at 37 °C for 16–20 h. Then, the absorbance was measured at 600 nm in a microplate reader (Epoch Biotek, Winooski, VT, USA). Ampicillin and sterile MH broth were used as a positive and negative control, respectively. The minimum inhibitory concentration (MIC) was defined as the lowest concentration of an antimicrobial where there was no visible growth. After measuring the MIC, a 50 μL sample was taken from the growth well without visible bacteria, serially diluted with phosphate-buffered saline (PBS, pH = 7.0), then spread on an MH agar solid medium and incubated at 37 °C for 12 h. The number of colonies was calculated, and the minimum drug concentrations that killed 99.9% of bacteria were considered the minimum bactericidal concentration (MBC) of the peptide.

### 4.6. Time-Killing Kinetics

Time-killing kinetics were determined with the method as previously described [30]. Hydrostatin-AMP2 (MBC) or Ampicillin (MBC) was co-incubated with bacterial suspension (*E. coli*) at 37 °C. The mixture was sampled 50 μL at 5, 15, 30, 60, 120, 240, and 480 min. Dilute the samples with sterile MH broth to a 10-fold gradient. Bacterial suspensions were plated at MH agar plates. After incubation at 37 °C for 12 h, the number of colonies was calculated.

### 4.7. Biofilm Inhibition Assay

The biofilm inhibition effect of Hydrostatin-AMP2 was determined with the method as previously described and little modified [30]. In 96-well plates, 100 μL of Hydrostatin-AMP2 (0.5-8 × MIC) in MH broth was incubated with 100 μL of the bacterial suspension (*S. aureus*) at 37 °C for 24 h. Sterile PBS was used to wash 3 times and methanol was used to fix it for 0.5 h. After drying, 1% crystal violet solution was used to stain for 0.5 h, washed 3 times with sterilized deionized water, and dissolved with 100 μL anhydrous ethanol. The absorbance was measured at 600 nm.

### 4.8. Biofilm Eradication Assay

The biofilm eradication effect of Hydrostatin-AMP2 was determined with the method as previously described and with little modification [42]. In 96-well plates, 100 μL of bacterial suspension (*S. aureus*) was seeded and incubated at 37 ℃ for 24 h. Wash well with sterile PBS 3 times. 100 μL of Hydrostatin-AMP2 (0.5–8 × MIC) or MH broth was added. After 24 h incubation, wash well with sterile PBS 3 times. Methanol was added to fix for 0.5 h and dried aseptically. 1% crystal violet solution was added to the stain for 0.5 h and washing 3 times with sterilized deionized water. Anhydrous ethanol dissolved biofilm. The absorbance was measured at 600 nm.

### 4.9. Induction of Resistance

Induction of resistance was determined across the change of MIC using a method as previously described with minor modifications [43]. *E. coli* was inoculated into MH broth and oscillated (37 °C, 220 rpm, 12 h) to the logarithmic growth stage, and then diluted to 10^6^ CFU/mL. With the absence or presence of 0.5×MIC Hydrostatin-AMP2 or Ampicillin, each panel culture oscillated for 12 h. MIC was detected and the previous process was repeated. Every 5 generations, the strain was frozen.

### 4.10. Salt Stability Analysis

The structural stability of Hydrostatin-AMP2 in salt solution (NaCl or CaCl_2_) was determined across the change of MIC by the method as previously described with minor modifications [44]. *E. coli* was inoculated into MH broth and oscillated (37 °C, 220 rpm, 12 h) to the logarithmic growth stage. Then diluted to 10^6^ CFU/mL in MH broth with NaCl (0, 50, 100, 150, and 200 mM) or CaCl_2_ (1, 2, and 4 mM). Hydrostatin-AMP2 was prepared from MH broth containing the corresponding concentration of NaCl or CaCl_2_. 100 μL Hydrostatin-AMP2 was mixed with 100 μL bacterial dilution in a 96-well plate, and incubated at 37 °C for 16–20 h. The absorption value was detected at 600 nm.

### 4.11. Thermal Stability Analysis

The structural stability of Hydrostatin-AMP2 at different temperatures was determined across the change of MIC. *E.coli* was inoculated into MH broth and oscillated (37 °C, 220 rpm, 12 h) to the logarithmic growth stage. Then diluted to 10^6^ CFU/mL. Hydrostatin-AMP2 was incubated at different temperatures (4, 37, 80, and 100 °C) for 1 h. In the 96-well plates, 100 μL of Hydrostatin-AMP2 (1–256 μg/mL) in MH broth was incubated with 100 μL of the bacterial suspension at 37 °C for 16–20 h. Then, the absorbance was measured at 600 nm.

### 4.12. Serum Stability Analysis

The structural stability of Hydrostatin-AMP2 in serum solution was determined across the change of MIC. Hydrostatin-AMP2 was dissolved in sterilized deionized water to a final concentration of 10 mg/mL. Human serum was mixed with Hydrostatin-AMP2 solution at a volume ratio v:v = 4:1 and incubated in a 37 °C incubator for 1, 2, 3, and 4 h, respectively. The same amount of solution was taken at each time point and gradient dilution was carried out with normal saline. In the 96-well plates, 100 μL of Hydrostatin-AMP2 (1–256 μg/mL) in MH broth was incubated with 100 μL of the bacterial suspension at 37 °C for 16–20 h. Then, the absorbance was measured at 600 nm.

### 4.13. pH Stability Analysis

*E. coli* was inoculated into MH broth and oscillated (37 °C, 220 rpm, 12 h) to the logarithmic growth stage. Then diluted to 10^6^ CFU/mL. Hydrostatin-AMP2 was incubated with PBS (pH = 2 or 12) at different pH values for 4 h at 37 °C. The MICs of these peptides were then measured.

### 4.14. Cytotoxicity Assays

The cytotoxicity of Hydrostatin-AMP2 against L929, RAW264.7, and HaCaT was determined by the CCK-8 method as previously described [45]. The cells were cultured using a DMEM medium containing 10%FBS and 1% penicillin-streptomycin solution. In 96-well plates, 100 µL of cells (2 × 10^4^ cells/well) in DMEM were seeded and cultured overnight in an incubator at 5% CO_2_ and 37 °C. Then, the culture medium was replaced by 100 μL of Hydrostatin-AMP2 in DMEM and cultured for 24 h. Subsequently, the medium was replaced by 100 μL DMEM containing 10% Cell Counting Kit-8 reagent, and incubated for 0.5 h at 37 °C. The absorbance was measured at 480 nm.

### 4.15. Hemolysis Assays

The hemolysis activity of Hydrostatin-AMP2 was determined using human erythrocytes, as previously described [46,47]. Normal saline was used to prepare 2% red blood cell suspension, and serial-diluted Hydrostatin-AMP2 solution was co-incubated with the erythrocytes at 37 °C for 0.5 h. Centrifuge at 25 °C at 1500 rpm for 5 min. Transfer 180 μL of supernatant to a 96-well plate and measure absorbance value at 540 nm. Normal saline was used as a negative control and 1% Triton X-100 solution as a positive control (complete hemolysis).

### 4.16. RNA Extract and Real-Time PCR

Mouse RAW264.7 cells were cultured in DMEM medium (containing 10% FBS, 1% penicillin-streptomycin solution). RAW264.7 were seeded in 6-well plates (2 × 10^6^ cells/well) and incubated in an incubator at 5% CO_2_ and 37 °C overnight [48,49]. Then, cells were incubated for another 2 h in the absence of a penicillin-streptomycin solution. After 2 h, 100 ng/mL LPS and Hydrostatin-AMP2 (5–20 μg/mL) were added in the medium at the same time and the cells were incubated for another 6 h at 37 °C.

Total RNA was extracted from the cell using RNAiso Plus and chloroform, and then the total RNA concentration and purity were measured using a NanoDrop 2000 spectrophotometer. cDNA was synthesized using the PrimeScript™ RT Master Mix. Real-time PCR was conducted using TB Green^®^ Premix Ex Taq™ II, and the reaction solution contained 5 μL TB Green^®^ Premix Ex Taq™ II, 0.2 μL of each primer (10 μM), 1 μL cDNA, and nuclease-free water (total volume 10 μL). The following primer sequences of glyceraldehyde 3-phosphate dehydrogenase (GAPDH), interleukins (IL-6, IL-1β, IL-10, IL-8), tumor necrosis factor-alpha (TNF-α), interferon-gamma (IFN-γ), monocyte chemoattractant protein-1 (MCP-1) and inducible nitric oxide synthase (iNOS), are presented in Table 4.

### 4.17. Statistical Analysis

Data were presented as means ± standard errors of mean. The graphs were drawn by software Prism version 6.01 (GraphPad, San Diego, CA, USA). All experiments was performed thrice.

## Figures and Tables

**Table 1:** Antimicrobial activity of Hydrostatin-AMP2.

	Microorganisms	MIC/MBC (μg/mL)	
		Hydrostatin-AMP2	Ampicillin
G^−^	*Escherichia coli*	16/32	8/16
	*Klebsiella pneumoniae 44*	32	>128
	*K. pneumoniae 48*	16	>128
	*K. pneumoniae 49*	32	>128
	*K. pneumoniae 50*	32	>128
	*K. pneumoniae 51*	32	>128
G^+^	*Propionibacterium acnes*	32	0.25
	*Staphylococcus aureus*	32	0.25

**Table 2:** Effects of salts, pH, serum, and temperature on the antimicrobial activity of Hydrostatin-AMP2.

Conditions		MIC (μg/mL) ^a^	Conditions		MIC (μg/mL)
	Control	16			
NaCl	50 mM	32	Human serum	1 h	32
	100 mM	32		2 h	32
	150 mM	32		3 h	32
	200 mM	64		4 h	32
CaCl_2_	1 mM	64	Temperature	4 °C	32
	2 mM	64		37 °C	32
	4 mM	128		80 °C	32
				100 °C	64
pH	2	64			
	12	64			

^a^*E. coli* was used to detect the antimicrobial activity of the peptide. The results represent the mean values of three independent experiments performed in triplicate.

**Table 3:** Cytotoxicity and hemolysis of Hydrostatin-AMP2.

Cells	Cell Death/Hemolysis (%) ^a^
L929	8.08
HaCaT	5.89
RAW264.7	10.81
Erythrocytes	4.6

^a^ The results represent mean values of three independent experiments performed in triplicate.

**Table 4:** Primer sequences (mouse) of inflammatory factors.

Gene	Forward Primer	Reverse Primer
GAPDH	5′-AGGTCGGTGTGAACGGATTTG-3′	5′-TGTAGACCATGTAGTTGAGGT-3′
IL-6	5′-CCAATGCTCTCCTAACAGAT-3′	5′-TGTCCACAAACTGATATGCT-3′
iNOS	5′-GCCAGTCAGGTCTCAGCAAG-3′	5′-CGCATGCAATGTGTGCTTGT-3′
IL-1β	5′-CACTACAGGCTCCGAGATGAACAAC-3′	5′-TGTCGTTGCTTGGTTCTCCTTGTAC-3′
TNF-α	5′-CACCACGCTCTTCTGTCTACTGAAC-3′	5′-AGATGATCTGAGTGTGAGGGTCTGG-3′
IFN-γ	5′-CTGGAGGAACTGGCAAAAGGATGG-3′	5′-GACGCTTATGTTGTTGCTGATGGC-3′
IL-10	5′-TGCCAAGCCTTATCGGAAATGATCC-3′	5′-AGCCGCATCCTGAGGGTCTTC-3′
MCP-1	5′-CACTCACCTGCTGCTACTCATTCAC-3′	5′-CTTCTTTGGGACACCTGCTGCTG-3′
IL-8	5′-CTTCTTTGGGACACCTGCTGCTG-3′	5′-CTTCTTTGGGACACCTGCTGCTG-3′

## Data Availability

Not applicable.
